# Rare Leiomyoma of the Tunica Dartos: A Case Report with Clinical Relevance for Malignant Transformation and HLRCC

**DOI:** 10.1155/2016/6471520

**Published:** 2016-07-27

**Authors:** Robert C. Bell, Evan T. Austin, Stacy J. Arnold, Frank C. Lin, Jonathan R. Walker, Brandon T. Larsen

**Affiliations:** ^1^Department of Pathology, University of Arizona, 1501 N. Campbell Avenue, Tucson, AZ 85724, USA; ^2^Division of Urology, Department of Surgery, University of Arizona, 1501 N. Campbell Avenue, Tucson, AZ 85724, USA

## Abstract

*Background*. Genital leiomyomas fall under the broader category of cutaneous leiomyomas, which are rare smooth muscle neoplasms accounting for 5% of all leiomyomas. Genital leiomyomas arising from the dartos muscle are exceedingly rare with fewer than 30 cases reported in the literature. They are typically benign and adequately treated with simple surgical excision; however, previously reported cases of malignant transformation and a possible link to the hereditary leiomyomatosis and renal cell cancer (HLRCC) syndrome warrant closer follow-up.* Case Presentation*. We report a case of a 47-year-old male refugee from Rwanda found to have a mobile, pea-sized, mildly painful scrotal lesion near the left penoscrotal junction and 1.5 cm indeterminate vascular mass in the right kidney. Surgical excision of the scrotal nodule was performed and the diagnosis of a dartoic leiomyoma was rendered. The presence of moderate nuclear atypia, rare mitotic activity, and close surgical margins prompted a wide reexcision. We report the surgical approach, pathologic findings, and clinical follow-up related to this scrotal lesion.* Conclusion*. Scrotal leiomyomas demonstrate a high rate of recurrence and pose a risk for malignant transformation. They may also indicate an underlying autosomal dominant syndrome associated with increased risk for development of an aggressive form of renal cell carcinoma. When discovered, management should include surgical excision, screening for syndromic features, and routine follow-up.

## 1. Introduction

Leiomyomas are benign smooth muscle neoplasms commonly found in the uterus and gastrointestinal tract. Cutaneous leiomyomas are a subcategory of leiomyomas comprising less than 5% of the total number of these tumors. Within this category are genital leiomyomas, which can arise from the smooth muscle of the tunica dartos, areola, or vulva. Although leiomyomas can originate anywhere along the male genitourinary system, scrotal leiomyomas arising from the tunica dartos are exceptionally rare with fewer than thirty cases having been reported in the literature [1–3]. Here we detail the case of a 47-year-old man who initially presented with a mildly painful scrotal lesion confirmed by pathologic evaluation to be a dartoic leiomyoma and discuss the potential future ramifications of this diagnosis to the patient.

## 2. Case Report

The patient is a 47-year-old male refugee from Rwanda who was referred for urologic evaluation by his primary care physician for a mildly painful, slowly enlarging nodule on his left hemiscrotum. The lesion had been present for two years, having become painful over the past 6 months. The patient reported no associated discharge or changes in color or texture. He denied any personal or family history of genitourinary malignancies or dermatologic issues.

A genitourinary examination demonstrated a mobile, pea-sized, scrotal lesion near the left penoscrotal junction. No other masses, lesions, or inguinal adenopathy were noted. At the time, the lesion appeared to be most consistent with a sebaceous cyst. A complete dermatologic examination was also performed which did not demonstrate any other lesions.

**Figure 1:**
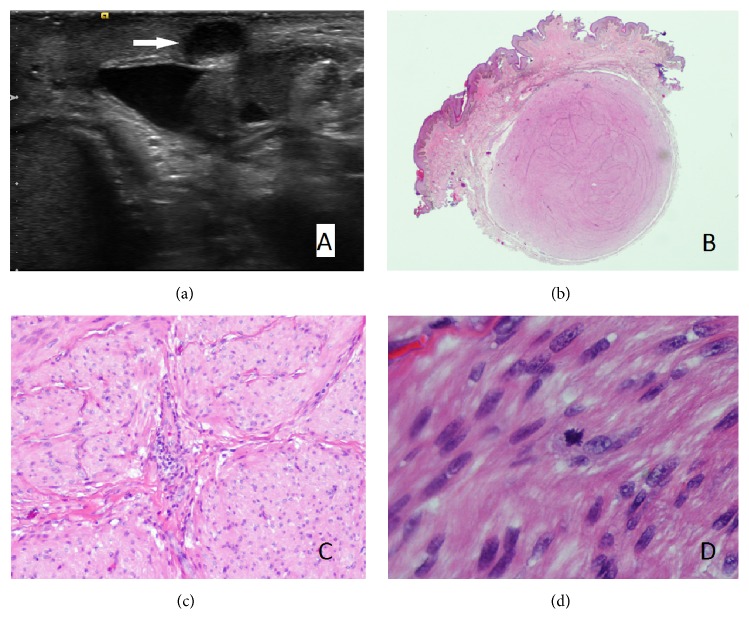
(a) Ultrasound imaging demonstrating hypoechoic mass at left penoscrotal junction. (b) Subcutaneous, unencapsulated, but well-circumscribed scrotal lesion with unremarkable overlying epidermis (H&E ×5). (c) Interlacing fascicles and bundles of smooth muscle with eosinophilic cytoplasm and elongate, cigar-shaped nuclei (H&E ×100). (d) Rare mitotic figure (H&E ×400).

Given the unknown etiology of this scrotal lesion, a scrotal ultrasound was performed to evaluate any deeper involvement. This demonstrated a 7 mm superficial, hypoechoic soft-tissue nodule (Figure 1(a)). An incidental right-sided varicocele and hydrocele and small bilateral epididymal cysts were also found. Of note, unilateral right-sided varicoceles are rare and may signify underlying pathology causing inferior vena caval obstruction, classically from tumor thrombus involving the IVC [4]. To further evaluate this right-sided varicocele, a renal ultrasound was performed which demonstrated an indeterminate vascular mass measuring 1.5 cm in the right kidney and a 1 cm mass consistent with a Bosniak type II cyst in the left kidney.

Due to the uncertain nature of the scrotal nodule and the patient's report of bothersome pain, the patient was offered continued observation or surgical excision and elected for the latter. After infiltration of the area with 1% lidocaine, an elliptical skin excision was made encompassing the lesion measuring approximately 1.0 cm × 2.0 cm. This was then dissected free from the subcutaneous tissue, showing no obvious extension of the nodule into deeper tissue. The patient tolerated the procedure well and was able to return home in stable condition.

**Figure 2:**
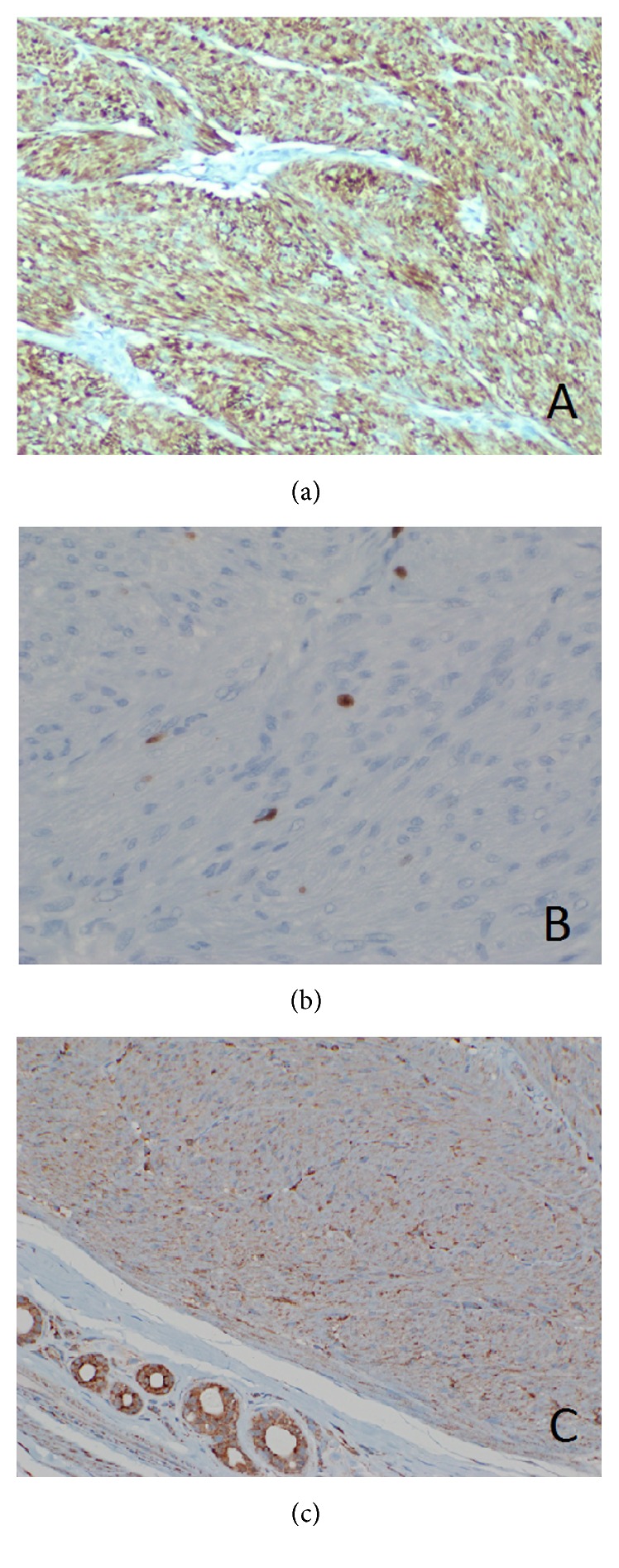
(a) Immunohistochemical staining for desmin shows strong, diffuse cytoplasmic staining of the lesional cells (×100). (b) Immunohistochemical staining for Ki-67 shows positivity in 1-2% of lesional nuclei (×400). (c) Immunohistochemical staining for fumarate hydratase, demonstrating intact expression of this enzyme in lesional cells (×200).

Gross evaluation of the specimen revealed a well-circumscribed, subcutaneous nodule measuring 0.8 cm. The nodule revealed homogenous, tan, whorled cut surfaces. Histologic evaluation revealed a well-circumscribed, nodular lesion arising from the tunica dartos, comprised of interlacing fascicles of bland, spindled cells with eosinophilic cytoplasm and blunt-ended, elongated, cigar-shaped nuclei with perinuclear halos and mild to moderate cytologic atypia (Figures 1(b) and 1(c)). Rare mitotic figures without atypia (0-1 per 10 high-power fields) were present (Figure 1(d)), without myxoid change or hyalinization. Lymphoid aggregates were present within the lesion, often in association with blood vessels. Immunohistochemical staining demonstrated immunoreactivity of the spindled cells using antibodies directed against smooth muscle actin and desmin, but not CD34, S-100 protein, or AE1/AE3, supporting a diagnosis of a smooth muscle neoplasm (Figure 2(a)). The proliferative index of the lesion, as demonstrated by a Ki-67 immunohistochemical stain, was 1-2% (Figure 2(b)).

After discussion of the pathology results with the patient including the close margin status of the original excision (less than 1 mm margin), the patient was offered observation or wide reexcision to prevent tumor recurrence. The patient wished to proceed with reexcision, which was performed without complication. Pathologic examination of the reexcision demonstrated chronic inflammation and fibrous tissue, consistent with a previous surgical site, but no residual leiomyoma. His follow-up two-week later examination demonstrated an appropriately healing surgical site.

The presence of a dartoic leiomyoma in this patient with a renal mass of undetermined etiology raised the possibility of hereditary leiomyomatosis and renal cell carcinoma syndrome (HLRCC). The underlying defect of this disorder is an inactivating germline mutation in fumarate hydratase (FH). Although not employed currently as a routine clinical screening method for HLRCC, we acquired an antibody directed against FH (J-13 fumarate hydratase, Santa Cruz Biotechnology, Santa Cruz, CA) and performed staining on the lesion using two benign uterine leiomyomas as positive controls. By this method, we found intact FH expression in the patient's lesion (Figure 2(c)) suggesting that the tumor most likely arose in a sporadic fashion and offering no overt evidence of FH deficiency or the HLRCC syndrome in the patient, although it should be remembered that FH immunohistochemistry is only 83% sensitive and 75% specific for HLRCC, and intact FH expression does not entirely exclude the possibility of HLRCC [5]. We also performed a triphasic contrasted CT scan to further characterize the possible right renal mass. This study, however, revealed no vascular, cystic, or solid abnormalities in the kidneys. MRI for follow-up may be optimal given the improved soft-tissue resolution and lack of ionizing radiation. Although no specific evidence of HLRCC syndrome was identified either clinically or pathologically and our results were reassuring, this remains a poorly understood syndrome and we believe that long-term surveillance for development of new or recurrent leiomyomas and renal tumors is still prudent.

## 3. Discussion

Cutaneous leiomyomas are rare tumors of smooth muscle origin that may arise in sporadic fashion or in the setting of HLRCC, a heritable tumor predisposition syndrome. Three subtypes of cutaneous leiomyoma are currently recognized, including piloleiomyomas (arising from pilar erector muscle), angioleiomyomas (arising from vascular smooth muscle), and genital leiomyomas [1, 2]. Genital leiomyomas are the most uncommon subtype and may originate from dartoic, vulvar, or mammary smooth muscle. Scrotal leiomyomas are most commonly found in Caucasian men, typically present in the fourth to sixth decade of life, and are initially painless although paroxysmal pain can develop over time [1, 2, 6].

Leiomyomas of the tunica dartos are typically well-circumscribed, unencapsulated nodules located in the subcutaneous tissue with a minimal to moderate amount of atypia [2, 3]. Genital leiomyomas can occasionally display a significant amount of nuclear atypia, which may represent a degenerative phenomenon and does not necessarily equate to increased risk for malignant behavior [3]. If left untreated, these tumors can continue to grow and even with surgical intervention, they may recur in up to 50% of cases [7]. Local recurrence has been reported over a wide range of time, from 6 weeks to greater than 15 years after excision [8]. Simple surgical excision is usually sufficient for treatment and further intervention such as radiotherapy should be avoided to limit risk of malignant transformation [1]. To minimize the likelihood of recurrence, it is recommended to ensure that all margins are clear of residual tumor [1, 2, 8].

Features suggestive of malignant transformation in these tumors include hemorrhage as well as diffuse cytologic/nuclear atypia, two or more mitotic figures per 10 high-power fields, and/or necrosis. As with all smooth muscle neoplasms of somatic soft tissues, distinction between cytologic atypia of malignancy and degenerative atypia can be problematic in clinical practice, although the suspicion for malignancy is increased when multiple ominous features are observed, particularly if necrosis is also present [8]. Cases of malignant transformation to leiomyosarcoma have been reported and some recommend closer monitoring for neoplasms demonstrating bizarre nuclei that do not appear degenerative in nature or neoplasms demonstrating increased mitotic activity [9, 10].

In an effort to describe the salient features and further define the diagnostic criteria of scrotal smooth muscle neoplasms, a recent comparative study by Matoso et al. reviewed 24 smooth muscle tumors of the scrotum. The case series included usual leiomyomas, symplastic leiomyomas, and leiomyosarcomas. In this study, the benign entities of usual and symplastic leiomyomas were small in size (less than 1.5 cm in diameter) and well-circumscribed, often contained lymphoid aggregates, and showed low proliferation indices (<5%) as demonstrated with Ki-67 staining with lack of significant mitotic activity. The level of atypia and increased cellularity were the main distinguishing features when making a distinction between usual and symplastic leiomyomas. The leiomyosarcomas, on the other hand, averaged 2.0 cm in diameter, were well-circumscribed with less ill-defined borders, did not contain lymphoid aggregates, and showed increased proliferative indices and mitoses [11]. While the presence of atypia and rare mitoses in our case raised the concern for leiomyosarcoma, a Ki-67 immunohistochemical stain demonstrated a very low proliferative index (1-2%), a reassuring finding more in keeping with a benign lesion. Overall, the small size (0.8 cm in diameter), mild to moderate atypia, minimal mitotic activity, and low Ki-67 proliferation index of our lesion are most consistent with a usual leiomyoma rather than a leiomyosarcoma.

Most cutaneous leiomyomas have unknown and sporadic pathogenesis. However, some occur in the setting of an underlying hereditary autosomal dominant tumor predisposition syndrome that is associated with increased risk for development of an aggressive form of renal cell carcinoma [7, 8]. Patients with the hereditary leiomyomatosis and renal cell cancer (HLRCC) syndrome may present with a wide range of clinical manifestations. Combinations include solitary or multiple leiomyomas, with or without renal tumors and/or uterine fibroids. In general, the presentation of cutaneous leiomyomas occurs earlier than renal tumors, with a median age of 25 years for the development of leiomyomas compared to a median age of 44 years for renal manifestations [12]. HLRCC is associated with a germline mutation in the gene coding fumarate hydratase, an enzyme that converts fumarate to malate in the Krebs cycle [8, 12]. Recent studies, including those by Llamas-Velasco et al. and Trpkov et al., have suggested that immunohistochemical testing for FH and its downstream product, S-(2succinyl) cysteine (2-SC), may be a viable screening method for HLRCC in cases of cutaneous leiomyomatosis and uterine leiomyomatosis and in certain high grade renal carcinomas, with results suggesting HLRCC (loss of FH and positive 2-SC) prompting more expensive confirmatory germline mutation testing [5, 13].

In patients with HLRCC syndrome, Malik et al. recommend annual full-body skin exams and biyearly radiological investigation in addition to surgical excision for evaluation and treatment of cutaneous leiomyomas [8]. Due to the limited reporting and rarity of this syndrome, there is limited epidemiologic data or analysis available, but we believe that these recommendations are reasonable at this time until more is known about this uncommon syndrome.

## 4. Conclusion

Genital leiomyomas of the scrotum are rare neoplasms. When this diagnosis is rendered, it is important to recognize that these neoplasms usually behave in a benign fashion but demonstrate a high rate of recurrence, can undergo malignant transformation in some cases, and may be the first clue suggesting a diagnosis of HLRCC. As such, it is important not only to ensure that the surgical margins are clear on initial excision but also to closely monitor the patient over time for the development of new or recurrent leiomyomas and/or renal tumors and to consider ancillary tests for HLRCC, such as FH immunohistochemistry and germline molecular testing.
